# Ulinastatin administration is associated with a lower incidence of acute kidney injury after cardiac surgery: a propensity score matched study

**DOI:** 10.1186/s13054-016-1207-7

**Published:** 2016-02-17

**Authors:** Xin Wan, Xiangcheng Xie, Yasser Gendoo, Xin Chen, Xiaobing Ji, Changchun Cao

**Affiliations:** Department of Nephrology, Nanjing First Hospital, Nanjing Medical University, 68 Changle Road, Nanjing, Jiangsu 210006 China; Department of Nephrology, Hangzhou First People’s Hospital, Nanjing Medical University, 261 Huansha Road, Hangzhou, Zhejiang 310006 China; Division of Thoracic and Cardiovascular Surgery, Nanjing First Hospital, Nanjing Medical University, Nanjing, Jiangsu China

**Keywords:** Acute kidney injury, Ulinastatin, Cardiac surgery, Cardiopulmonary bypass

## Abstract

**Background:**

Systemic inflammation is involved in the development of acute kidney injury (AKI) after cardiac surgery with cardiopulmonary bypass (CPB). Ulinastatin, a urinary trypsin inhibitor (UTI), possesses a variety of anti-inflammatory effects. Therefore, we hypothesized that the administration of ulinastatin would reduce the occurrence of AKI in patients undergoing cardiac surgery with CPB.

**Methods:**

A retrospective propensity score matched analysis was used to evaluate the effect of ulinastatin on the development of AKI in patients undergoing first documented cardiac surgery with CPB between January 2008 and December 2012 in our hospital. Multiple logistic regression models were also employed to identify the association between UTI administration and development of AKI.

**Results:**

A total of 2072 patients who underwent cardiac surgery with CPB met the inclusion criteria. Before propensity score matching, variables such as age, baseline creatinine, CPB duration, red blood cells transfused, and hematocrit were statistically different between the ulinastatin (UTI) group and the control group. On the basis of propensity scores, 409 UTI patients were successfully matched to the 409 patients from among those 1663 patients without UTI administration. After propensity score matching, no statistically significant differences in the baseline characteristics were found between the UTI group and the control group. The propensity score matched cohort analysis revealed that AKI and the need for renal replacement therapy occurred more frequently in the control group than in the UTI group (40.83 % vs. 30.32 %, *P* = 0.002; 2.44 % vs. 0.49 %, *P* = 0.02, respectively). However, there were no significant differences in mortality, length of intensive care unit stay, and length of hospital stay between the UTI group and the control group. Using multivariate logistic regression analysis, we found ulinastatin played a protective role in the development of AKI after cardiac surgery (odds ratio 0.71, 95 % confidence interval 0.56–0.90, *P* = 0.005).

**Conclusions:**

This study shows that ulinastatin was associated with a lower incidence of AKI after cardiac surgery, suggesting that the administration of ulinastatin may be favorable for those patients undergoing cardiac surgery with CPB.

## Background

Acute kidney injury (AKI) commonly occurs in patients undergoing cardiac surgery, especially those treated with cardiopulmonary bypass (CPB) [1]. The incidence of cardiac surgery–associated (CSA) AKI ranges from 8.9 % [2] to 39 % [3], depending on the definition used. Renal replacement therapy (RRT) is needed in 1–5 % of patients [4], and the mortality rate is 1.4 % [5]. Previous studies demonstrated that even mild elevation in serum creatinine (SCr) levels after cardiac surgery is significantly associated with higher mortality [6–8]. However, the treatment for CSA-AKI still remains challenging [9]; thus, prevention is the mainstay of effective treatment for patients at high risk of developing AKI.

There are multiple factors involved in the development of CSA-AKI, including hemodynamic, inflammatory, metabolic, and nephrotoxic factors [10]. Emerging evidence shows that systemic inflammation plays a key role in the development of CSA-AKI and participates in renal injury, especially tubular lesions [11–15].

To date, pharmacological renal protection strategies remain limited [16]. Ulinastatin, a urinary trypsin inhibitor (UTI) that inhibits various inflammatory proteases, including chymotrypsin, trypsin, and neutrophil elastase, has been widely used in China, Japan, and Korea for the treatment of patients with inflammatory disorders, postoperative organs protection, shock, and pancreatitis [17–19]. A previous study has shown that ulinastatin can decrease cytokine concentrations in patients undergoing cardiac surgery [20].

However, studies investigating its renoprotective role in the development of AKI in patients undergoing cardiac surgery with CPB are limited [21]. Thus, the aim of the present study was to test the hypothesis that the administration of ulinastatin would reduce the incidence of CSA-AKI.

## Methods

### Patients

This retrospective study included patients aged 18 years or older who underwent cardiac procedures with CPB at the Nanjing First Hospital in Nanjing, China, between January 2008 and December 2012. A total of 2072 patients were selected for analysis. Patients with end-stage kidney disease needing RRT before surgery were excluded.

This study was performed in accordance with the Declaration of Helsinki and was approved by the Regional Human Research Ethics Committee of Nanjing First Hospital (reference 201409–1). Individual patient consent was waived on condition that all patient data were deidentified before analysis, because this study was a retrospective analysis. The most recent examined SCr value within 7 days before surgery was defined as the baseline creatinine level.

### Anesthesia

Patients were premedicated with intramuscular morphine 0.2 mg/kg and scopolamine 0.3 mg. Anesthesia induction was performed with midazolam 0.2 mg/kg, fentanyl 10 μg/kg, and vecuronium 0.15 mg/kg, and then tracheal intubation was performed. Anesthesia was maintained with continuous propofol (4–8 mg/kg/h) and intermittent intravenous fentanyl, midazolam, vecuronium, and inhalation of 1–2 % isoflurane. Right internal jugular vein was cannulated for transfusion. Electrocardiography, central venous pressure, mean arterial pressure (MAP), arterial oxygen saturation, and nasopharynx and rectal temperature were monitored.

### Ulinastatin administration

Patients who were prescribed with and without ulinastatin were identified and correspondingly divided into the UTI group and the control group. Patients in the UTI group received a bolus dose of ulinastatin 500,000 KIU intravenously in 50 ml of saline for 15 minutes after induction of anesthesia.

### Anticoagulation

The dose of unfractionated heparin via central venous catheter used for anticoagulation during CPB was 300–400 U/kg plus additional doses to achieve and maintain an activated clotting time between 480 and 600 seconds. Protamine sulfate was used to reverse heparin-induced anticoagulation after separation from CPB.

### CPB management and surgical procedures

The CPB circuit was primed with a solution containing 500 ml of crystalloid solution, 1000 ml of hydroxyethyl starch, and 200 ml of 20 % mannitol. Management of CPB included alpha-stat pH management, MAP in the range of 50–80 mmHg, hematocrit of 20–25 %, and a non-pulsatile flow rate of 2.0–2.4 L/min/m^2^. The surgical procedures included coronary artery bypass grafting, mitral valve replacement, mitral and aortic valve replacement, and aortic valve replacement.

### Data collection

The demographic, preoperative, intraoperative, and postoperative data were collected from an electronic medical record database. SCr was recorded each day until the seventh day after surgery. Urine output data were not collected, owing to absence of these data in the records.

### Outcome endpoint

The primary endpoints were set as CSA-AKI, which was defined using Kidney Disease: Improving Global Outcomes criteria as an increase in SCr ≥0.3 mg/dl (≥26.5 μmol/L) within 48 h, and an increase in SCr to ≥1.5 times baseline known or presumed to have occurred within the prior 7 days. Overall mortality, need for RRT, intensive care unit (ICU) length of stay, and hospital length of stay were also recorded.

### Assessment of adverse events

Adverse events associated with ulinastatin, such as nausea, vomiting, leukocytopenia, hypersensitivity reactions, and elevation of transaminase, were recorded.

### Statistical methods

The data were analyzed using SPSS version 18.0 software (SPSS, Chicago, IL, USA) and the MatchIt package in R (version 2.8.1; http://www.r-project.org/). Continuous variables following a normal distribution were expressed as mean ± standard deviation, and categorical variables were expressed as percentages. An unpaired *t* test was employed to compare means between two groups. The χ^2^ test or Fisher’s exact test was used to compare categorical variables between two groups of subjects. The Mann–Whitney *U* test was used to compare medians. Multiple regression binary logistic regression with the backward stepwise method was performed to evaluate the relationship between the administration of ulinastatin and the occurrence of AKI. The significant acceptance and removal levels for a covariate were set at 0.05 and 0.1, respectively. Data were listed as odds ratios (ORs) with 95 % confidence intervals (CIs). Adjusted variables were age, sex, body mass index (BMI), history of hypertension, history of diabetes, insulin-controlled diabetes, chronic obstructive pulmonary disease (COPD), chronic kidney disease, cerebrovascular disease, MAP, history of coronary angiography, ejection fraction, preoperative baseline creatinine level, hematocrit, red blood cells (RBCs) transfused, CPB duration, body temperature (>38 °C) after surgery within 3 days, and mechanical ventilation time.

### Propensity score matching

Propensity scores for each subject were generated using a multivariable logistic regression analysis model to compute the probability of ulinastatin administration based on the following covariates: age, sex, BMI, diabetes, insulin-controlled diabetes, hypertension, COPD, chronic kidney disease, cerebrovascular disease, coronary angiography, preoperative baseline creatinine level, CPB duration, MAP, erythrocyte transfusion, ejection fraction, history of coronary angiography, hematocrit, and mechanical ventilation. Propensity scores were then employed to create 1:1 matched pairs (matching the UTI users to non-UTI users) using a nearest neighbor matching algorithm without a caliper method.

## Results

### Patient characteristics

**Fig. 1:**
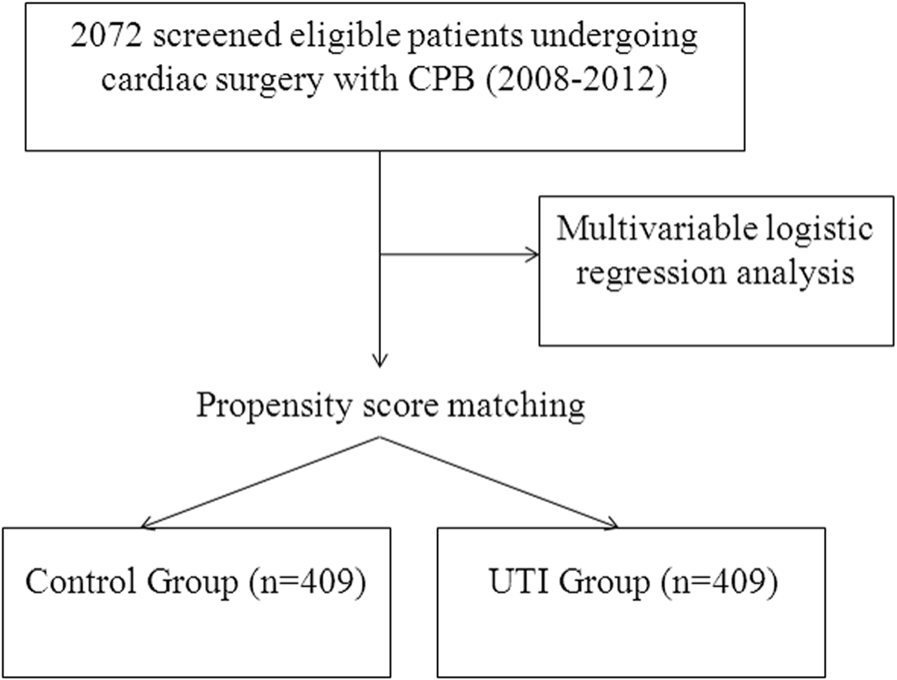
Flowchart showing patients included in the analysis. *CPB* cardiopulmonary bypass, *UTI* urinary trypsin inhibitor

A total of 2072 patients who underwent cardiac surgery with CPB met the inclusion criteria. Characteristics of the study subjects before and after propensity score matching are listed in (Table 1). Age, baseline creatinine, CPB duration, RBCs transfused, and hematocrit were statistically different between the UTI and control groups. On the basis of the propensity score, 409 patients who received UTI were successfully matched to 409 patients who did not have the UTI treatment (Fig. 1). After propensity score matching, no statistically significant baseline characteristics between the UTI group and the control group were found.

**Table 1:** Baseline characteristics of subjects before and after propensity score matched analysis

Variable	Before matching			Propensity score matched		
	Control group (*n* = 1663)	UTI group (*n* = 409)	*P* value	Control group (*n* = 409)	UTI group (*n* = 409)	*P* value
Age, yr	56 ± 13	54 ± 14	0.008	54 ± 14	54 ± 14	0.880
Male sex, *n* (%)	883 (53)	234 (57)	0.135	235 (57.45)	234 (57.21)	0.944
BMI, kg/m^2^	23.6 ± 3.6	23.4 ± 3.6	0.315	23.5 ± 3.7	23.4 ± 3.6	0.757
History of hypertension, *n* (%)	581 (36)	125 (31)	0.094	119 (29.1)	125 (30.6)	0.647
History of diabetes mellitus, *n* (%)	175 (10.5)	40 (9.8)	0.315	44 (10.8)	40 (9.8)	0.363
Insulin-controlled diabetes, *n* (%)	99 (6.0)	26 (6.4)	0.563	27 (6.6)	26 (6.4)	0.887
COPD, *n* (%)	27 (1.6)	11 (2.7)	0.150	9 (2.2)	11 (2.7)	0.490
Chronic kidney disease, *n* (%)	30 (1.8)	5 (1.2)	0.414	3 (0.73)	5 (1.2)	0.722
Cerebrovascular disease, *n* (%)	80 (4.8)	15 (3.7)	0.322	14 (3.4)	15 (3.7)	0.850
Coronary angiography, *n* (%)	590 (35.5)	150 (36.7)	0.651	165 (40.3)	150 (36.7)	0.281
Ejection fraction, %	59.2 ± 8.4	59.1 ± 9.4	0.953	58.9 ± 8.7	59.1 ± 9.4	0.761
Creatinine, μmol/L	75.0 ± 33.3	82.4 ± 26.9	<0.001	82.9 ± 53.9	82.4 ± 26.9	0.889
CPB duration, min	112.9 ± 48.0	106.3 ± 42.0	0.006	103.2 ± 40.2	106.3 ± 42.0	0.285
MAP, mmHg	62.3 ± 7.3	62.1 ± 5.6	0.515	62.2 ± 7.8	62.1 ± 5.6	0.803
RBCs transfused, U	4.3 ± 4.2	5.8 ± 4.9	<0.001	5.3 ± 5.3	5.8 ± 4.9	0.241
Hematocrit, %	23.4 ± 4.6	25.0 ± 5.6	<0.001	25.1 ± 5.3	25.0 ± 5.6	0.736
Mechanical ventilation, median (range)	7.8 (5.5–10.6)	7.8 (5.9–11.2)	0.073	7.8 (5.2–11.7)	7.8 (5.9–11.2)	0.078

*Abbreviations*: *BMI* body mass index, *CPB* cardiopulmonary bypass, *MAP* mean arterial pressure, *COPD* chronic obstructive pulmonary disease, *UTI* urinary trypsin inhibitor, *RBCs* red blood cells

### Comparison of patient outcomes

**Fig. 2:**
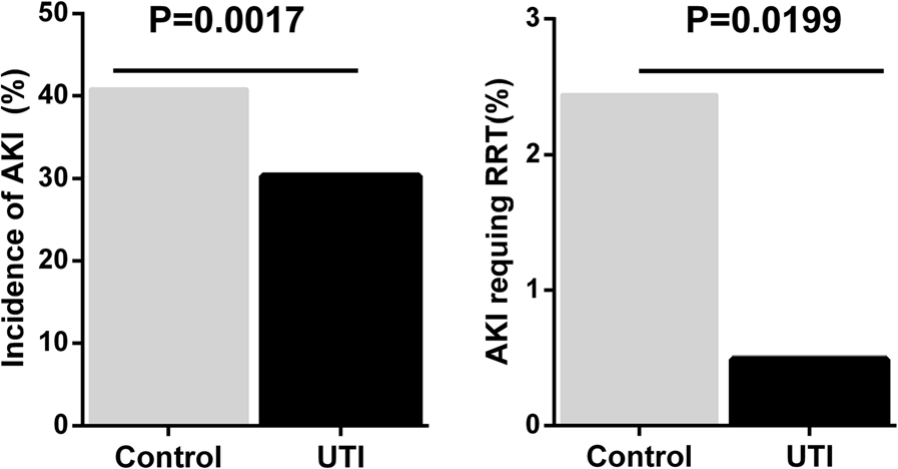
Incidence of acute kidney injury (AKI) and AKI requiring renal replacement therapy between the control group and urinary trypsin inhibitor (UTI) group

Cohort analysis revealed that AKI and RRT occurred more frequently in the control group (40.83 % vs. 30.32 %, *P* = 0.002; 2.44 % vs. 0.49 %, *P* = 0.02, respectively) (Fig. 2). No statistically significant differences in ICU length of stay, in-hospital length of stay, and mortality were found between the control group and the UTI group (*P* > 0.05) (Table 2).

**Table 2:** Comparison of outcomes between the control group and the UTI group (propensity score matching)

Outcome	Control group (*n* = 409)	UTI group (*n* = 409)	*P* value
AKI, *n* (%)	167 (40.83)	124 (30.32)	0.002
RRT, *n* (%)	10 (2.44)	2 (0.49)	0.020
Death, *n* (%)	11 (2.69)	4 (0.98)	0.068
Length of ICU stay, d	2.5 ± 7.1	2.0 ± 2.09	0.161
Length of in-hospital stay d	22.5 ± 10.3	21.6 ± 7.4	0.147

*Abbreviations*: *AKI* acute kidney injury, *RRT* renal replacement therapy, *UTI* urinary trypsin inhibitor, *ICU* intensive care unit

### Ulinastatin administration is a protective factor in the development of AKI

Multivariate logistic regression analysis was used to determine the possible protective role of ulinastatin in the development of AKI. The results are listed in Table 3. Notably, the administration of ulinastatin was found to be beneficial for protecting against CSA-AKI development (OR 0.71, 95 % CI 0.56–0.90, *P* = 0.005). The independent risk factors for CSA-AKI were as follows: male sex (OR 1.36, 95 % CI 1.12–1.66, *P* = 0.002), BMI (OR 1.28, 95 % CI 1.10–1.48, *P* = 0.001), history of hypertension (OR 1.58, 95 % CI 1.23–1.96, *P* < 0.001), insulin-dependent diabetes mellitus (OR 1.59, 95 % CI 1.07–2.35, *P* = 0.021), CPB duration ≥110 minutes (OR 1.38, 95 % CI 1.13–1.68, *P* = 0.001), body temperature (>38 °C) after surgery within 3 days (OR 1.22, 95 % CI 1.016–1.49, *P* = 0.044), RBC units transfused (OR 1.09, 95 % CI 1.06–1.12, *P* < 0.001), and mechanical ventilation time ≥9 h (OR 1.01, 95 % CI 1.00–1.02, *P* = 0.007).

**Table 3:** Multivariable analysis determining covariate factors associated with AKI development

Variable	Adjusted OR	95 % CI	*P* value
Male sex	1.36	1.12–1.66	0.002
BMI	1.28	1.10–1.48	0.001
History of hypertension	1.58	1.23–1.96	<0.001
Insulin-dependent diabetes	1.59	1.07–2.35	0.021
RBCs transfused, U	1.09	1.06–1.12	<0.001
CPB duration ≥110 min	1.38	1.13–1.68	0.001
Mechanical ventilation ≥9 h	1.01	1.00–1.02	0.007
Body temperature (>38 °C) after surgery within 3 days	1.22	1.01–1.49	0.044
Ulinastatin administration	0.71	0.56–0.90	0.005

*Abbreviations*: *AKI* acute kidney injury, *OR* odds ratio, *CI* confidence interval, *BMI* body mass index, *RBCs* red blood cells, *CPB* cardiopulmonary bypass

### Assessment of adverse events

No adverse events associated with UTI were found.

## Discussion

In the present study, we investigated the role of ulinastatin in the occurrence of AKI in patients undergoing cardiac surgery with CPB. Using a propensity score matching method, we found that patients receiving UTI treatment had a significantly lower incidence of AKI than those in the control group (30.32 % vs. 40.83 %, *P* = 0.002). Multivariate logistic regression analysis also revealed that ulinastatin played a beneficial role in the development of AKI. The independent risk factors found in our study, such as history of hypertension, insulin dependent diabetes, mechanical ventilation, CPB duration, and erythrocyte transfusion, were in accord with a previous study [9], while early postoperative fever (>38 °C in the first 72 h), which is rarely caused by an infection [22], was found for the first time (to the best of our knowledge) to be an independent risk factor for CSA-AKI. The underlying mechanism was thought to be related to systemic inflammatory responses, which is one of the postoperative complications in patients undergoing cardiac surgery with CPB [23]. These findings may be of importance in light of the renal protection in this setting due to CSA-AKI being significantly associated with higher postoperative mortality [8].

Systemic inflammatory response syndrome caused by cardiac surgery was found to play a central role in the development of AKI [24, 25] by deteriorating ischemia-reperfusion injury, resulting in tubular epithelial and vascular endothelial injury [11]. A large randomized clinical trial has demonstrated that patients undergoing cardiac surgery with CPB are more likely to have CSA-AKI than those treated with surgery using a beating-heart (off-pump) technique [1]. The possible mechanism may be associated with systemic inflammatory response, which occurs more frequently in patients who undergo CPB than in those treated with off-pump surgery [26]. The pathogenesis of systemic inflammatory responses involves multiple factors, such as surgical trauma, blood loss, transfusion, hypothermia, activation of the immune system, ischemia-reperfusion injury and endotoxemia [27]. The activation of humoral and cellular cascades results in an increase of proinflammatory cytokines, and eventually leads to cellular damage and organ injury [28].

Currently, the prophylactic pharmacologic agents aimed at attenuating the inflammatory response mainly include propofol, statins, and glucocorticoids [29]. The benefits of using corticosteroid prophylaxis as an anti-inflammatory agent in patients undergoing cardiac surgery with CPB remain controversial [30]. Although evidence indicates that corticosteroids are effective in reducing the risk of atrial fibrillation and mechanical ventilation duration [31], no difference in major outcomes was found, such as myocardial infarction, renal failure, and postoperative 30-day mortality [32, 33].

In the clinical practical setting, steroids should be prescribed cautiously for those patients with contraindications to steroids, such as peptic ulcer, diabetes mellitus, infection, and fracture. In contrast to steroids, ulinastatin, a powerful protease inhibitor derived from human urine, has unique anti-inflammatory properties, which include inhibition of neutrophil elastase and other proteases and suppression of the production of cytokines, such as interleukin (IL)-6, IL-8, and tumor necrosis factor-α, as well as endothelial leukocyte adhesion molecule-1 [34–38]. The possible side effects of ulinastatin, such as digestive symptoms, leukocytopenia, and hypersensitivity reactions, have rarely been observed in clinical studies [39–41].

On the basis of these properties of ulinastatin, it has been used to prevent postoperative complications and post–pump organ injury in patients undergoing cardiac surgery with CPB [42]. Nakanishi and coworkers [43] demonstrated that prepump administration of ulinastatin can suppress the elevation of postoperative IL-6 and IL-8 in patients undergoing coronary artery bypass graft surgery with CPB in a prospective, randomized, double-blind, placebo-controlled study. A meta-analysis of randomized controlled trials also indicates that ulinastatin can significantly decrease cytokine concentrations in patients undergoing cardiac surgery compared with those who received placebo [44]. Of note, a study of 60 subjects in Korea showed that ulinastatin administration has no cardiac or renal protective effects in patients undergoing aortic valve replacement with CPB [45]. In the study, the sample size of 30 patients in the UTI group and 30 patients in the control group was relatively small. Furthermore, the observation time points were only postoperative day 1 and day 2. The mean levels of SCr, cystatin C, and neutrophil gelatinase-associated lipocalin were employed to determine renal injury was present, instead of using the generally accepted Acute Kidney Injury Network or RIFLE (risk, injury, failure, loss, and end-stage kidney disease) criteria, making the study not so convincing. In addition, a letter to the editor in the same journal also raised concerns that the result should be interpreted with caution due to the relatively small sample size and the multifactorial causes of AKI [46].

Our data show that the administration of ulinastatin during CPB played a protective role in reducing the risk of AKI after cardiac surgery. Although the ICU and hospital lengths of stay seemed longer and mortality in the control group was higher, no statistically significant differences were found between the two groups. One possible reason is that the postoperative patients admitted to the ICU were then transferred to common wards on the second day as routine practice, unless the patients were in critical condition. Therefore, despite the fact that some patients developed AKI, a minor impact on the length of ICU stay resulted. The length of in-hospital stay was also influenced by the policy in our hospital, which limited the average length of hospital stay. Another aspect is that multiple factors including all kinds of complications, such as low cardiac output syndrome, bleeding, infection, and heart failure, can affect mortality and length of hospital stay. Further prospective randomized controlled trials with large sample sizes and perioperative administration of ulinastatin are warranted to confirm the protective role of ulinastatin in the development of CSA-AKI.

Our study has notable limitations. First, our study is a retrospective, single-center study, making it prone to bias. Second, due to the lack of urine output values, only creatinine was used to define AKI criteria. In addition, determining AKI on the basis of urine output was less than practical due to the urinary catheters’ usually being removed about 2 days after surgery.

## Conclusions

Our study shows that ulinastatin administration was associated with a lower incidence of CSA-AKI, suggesting that the administration of ulinastatin may be favorable for those patients undergoing cardiac surgery with CPB.

## Key messages

- Our results reveal ulinastatin administration was associated with a lower incidence of CSA-AKI using a propensity score methodology.
- Ulinastatin administration may play a protective role against the development of CSA-AKI in patients undergoing cardiac surgery with CPB.

## Abbreviations

AKI: acute kidney injury
BMI: body mass index
CI: confidence interval
COPD: chronic obstructive pulmonary disease
CPB: cardiopulmonary bypass
CSA-AKI: cardiac surgery–associated acute kidney injury
ICU: intensive care unit
IL: interleukin
MAP: mean arterial pressure
OR: odds ratio
RBC: red blood cell
RRT: renal replacement therapy
SCr: serum creatinine
UTI: urinary trypsin inhibitor
